# Application of survival analysis to model proliferation likelihood of *Escherichia coli* biofilm following laser-induced hyperthermia treatment

**DOI:** 10.3389/fbioe.2023.1001017

**Published:** 2023-01-24

**Authors:** Bradley Burden, Juan Sebastian Rodriguez-Alvarez, Nicole Levi, F. Scott Gayzik

**Affiliations:** ^1^ Department of Biomedical Engineering, Wake Forest University School of Medicine, Winston-Salem, NC, United States; ^2^ Department of Plastic and Reconstructive Surgery, Wake Forest University School of Medicine, Winston-Salem, NC, United States

**Keywords:** biofilm, hyperthermia, survival analysis, silicone, nanocomposite, photothermal

## Abstract

Eighty percent of bacterial infections associated with living tissue and medical devices are linked to drug-resistant biofilms, leading to lengthy and costly recoveries. Laser-induced hyperthermia can disrupt cell proliferation within biofilms and increase susceptibility to antibiotics. However, there can be bacterial survival differences dependent upon laser irradiation times, and prolonged time at elevated temperature can damage healthy tissue. The objective of this study was to use survival analysis to model the impact of temperature increases on reducing viable biofilm bacteria. *In vitro* biofilms of *Escherichia coli* were grown on silicone discs or silicone doped with photothermal poly(3,4-ethylenedioxythiophene) hydrate (PEDOT) nanotubes, and subjected to laser-induced hyperthermia, using a 3 W continuous wave laser at 800 nm for varying times. The number of colony forming units per milliliter (CFU/mL) and maximum temperature were measured after each trial. Survival analysis was employed to estimate bacterial cell proliferation post-treatment to provide a quantitative framework for future studies evaluating photothermal inactivation of bacterial biofilms. The results demonstrate the first application of survival analysis for predicting the likelihood of bacterial cell proliferation based on temperature.

## 1 Introduction

Biofilm is a matrix containing proteins, polysaccharides, and microbial DNA whose development is initiated by planktonic, or “free moving,” bacteria (Høiby et al., 2010). Micro-colonies quickly form, and the biofilm matrix thickens, protecting bacteria from antibiotics and adverse conditions (Mah and O’Toole, 2001; Sharma et al., 2016; Wang et al., 2021). Chronic bacterial infections with biofilm-producing bacteria such as *E. coli* are characterized by tissue damage and continued inflammation (Bjarnsholt et al., 2009; Høiby et al., 2010). Although the general risk of infection is low at 1%–7%, 80% of hospital acquired infections are caused by biofilms associated with intravenous catheters and other implanted medical devices (Bryers and Ratner, 2004; Sandora and Goldmann, 2012; VanEpps and Younger, 2016; Beckwith et al., 2020). The mortality rates are device-dependent, ranging from 5% for dental implants to greater than 25% for heart valves (VanEpps and Younger, 2016). Both short- and long-term indwelling catheters are usually comprised of silicone, which is more easily infected than other materials (Sherertz et al., 1995; Marosok et al., 1996). When antibiotics fail, the only course of treatment is implant removal (Mah and O’Toole, 2001; Sharma et al., 2016). This option presents its own complications, as many devices are not easily replaced and can cause additional trauma (Richardson et al., 2016). Biofilm infections place a huge economic burden on the medical device industry and healthcare system. In 2001, treatment of biofilm infections cost over $6 billion annually in the United States, and in 2012 the average cost of one catheter-associated infection was over $70,000 (Bryers and Ratner, 2004; Gbejuade et al., 2015; Beckwith et al., 2020).

### 1.1 Hyperthermia

#### 1.1.1 Treatment

Hyperthermia has been explored as a treatment for bacterial infections since increased temperatures can disrupt bacterial membranes and protein synthesis (Pavlovsky et al., 2015; Ibelli et al., 2018; Beckwith et al., 2020). While most bacteria thrive between 33°C and 41°C, *E. coli*’s outer membrane may be disrupted at elevated temperatures (above 45°C), leading to irreversible damage to bacterial structural integrity and disruption of cell proliferation (Ibelli et al., 2018). This temperature is commonly used as a threshold between mild and ablative hyperthermia because this is where eukaryotic cells also begin to suffer irreparable damage (Ibelli et al., 2018; Payne et al., 2020).

A popular method for localized hyperthermia is photothermal therapy (PTT), which involves the application of light, most often near infrared (NIR) radiation, to a specific material that absorbs the light and generates heat (Ibelli et al., 2018). As the temperatures increase, irreversible damage triggers eukaryotic cells to undergo apoptosis and, beyond 60°C, tissue charring, protein denaturation, and necrosis occurs (Balivada et al., 2010; Brace, 2011; Lee et al., 2018; Payne et al., 2020). Ablation has been researched for oncological uses in the destruction of malignant tumors, but much remains unknown regarding its possibilities for treating bacterial infections (Beachy and Repasky, 2011; Brace, 2011). It is well established that bacteria can be inactivated by hyperthermia, with time as an important variable and the current work seeks to capitalize upon rapid photothermal inactivation of bacterial biofilms. Common photothermal materials include semiconducting polymers like polyethylene dioxythiophene (PEDOT), polypyrrole, and polyaniline (Abel et al., 2014; Xu et al., 2014; Li et al., 2015; Ruiz-Pérez et al., 2020; Zhang et al., 2022).

#### 1.1.2 Arrhenius-Eyring-Polanyi (AEP) model

The Arrhenius equation (Eq. 1) has been used in microbiology to estimate the effect of temperature on the inactivation rate of bacteria (Huang et al., 2011; Pandey and Soupir, 2011). The rate coefficient *k*, described as cell death rate (sec^−1^), is a function of temperature *T*_*abs*_ (K) that includes the universal gas constant *R* (8.314 J mol^−1^ K^−1^) and substance-dependent parameters *A*, a

$$k=Ae^{-\frac{\Delta E_{a}}{RT_{abs}}}$$ (1)

frequency factor (sec^−1^), and *ΔE*_*a*_, inactivation energy (J mol^−1^). One challenge of using the Arrhenius model for evaluating thermal inactivation of bacteria is the model becomes non-linear for some temperature regions, depending on bacterial species, and therefore fails as a predictive model (Mouratidis et al., 2019). To bypass this issue, Huang et al. (2011) modified Eq. 1 to create Eq. 2, the Arrhenius-Eyring-Polanyi (AEP) equation, by adding an additional temperature term and creating a regression coefficient, *A*_*AEP*_, and replacing the inactivation energy with a separate energy term, *ΔG*’ (J mol^−1^), and exponent *α* (Huang et al., 2011). Each of the remaining variables are

$$k=A_{AEP}T_{abs}e^{-{\left(\frac{\Delta G^{\prime }}{RT}\right)}^{\alpha }}$$ (2)

The same as Eq. 1. While this model is useful in recognizing thermal breakpoints in Arrhenius plots, its unique variables may be challenging to discern experimentally for bacteria (Huang et al., 2011).

#### 1.1.3 Utilization of the D/z-concept

While Arrhenius plots provide details about the relationship between cell death rate and temperature, they do not consider time-temperature history. To address this, the *D/z*-concept is commonly used for thermal sterilization/inactivation in the food industry to estimate log reductions of bacteria (Fujikawa and Itoh, 1998; van Asselt and Zwietering, 2006). This model assumes a log-linear inactivation to determine cell death rate (van Asselt and Zwietering, 2006). *D* is the time necessary (in minutes) to achieve a 1-log reduction of bacteria, or the time it takes to kill 90% (Miles et al., 1995; Juneja et al., 1997). These values are calculated from linear sections of experimental data by plotting the log of surviving bacteria versus heating times. The *z*-values are the temperature increases (°C) needed to reduce the corresponding *D*-value by a factor of 10 (Miles et al., 1995; van Asselt and Zwietering, 2006).

A range of *D*- and *z-*values have been determined based on thermal inactivation of bacteria. Research by van Asselt and Zwietering (2006) explored applying the *D/z*-concept to specific strains of bacteria to minimize experimental variance and more accurately predict cell death and improve thermal sterilization efficiency. This paradigm was used to determine a cell death rate, *k*_*m*_ (min^−1^), for *E. coli*, using Eqs 3, 4. *D*-values across varying temperatures and experiment procedures were obtained from literature to determine a reference value, *D*_*ref*_ (min). *T*_*ref*_ is a reference temperature (°C) in the range of the heating process dependent on the pathogen

$$\log\left(D\right)=\log\left(D_{ref}\right)-\frac{T-T_{ref}}{z}$$ (3)

$$D=\frac{2.303}{k_{m}}$$ (4)

(van Asselt and Zwietering, 2006). Once these reference values are determined, *D*-values can be calculated at any temperature.

### 1.2 Survival analysis

Survival analysis is a set of statistical methods that model when an event of interest occurs in relation to the measurement of a monotonically increasing independent variable. This process has been adopted in human injury probability, and is used in automotive, military, and sports applications (Petitjean et al., 2012; Yoganandan et al., 2016; Yoganandan and Banerjee, 2018; Hostetler et al., 2021). The event of interest in the current study is when enough cell death has occurred such that cell proliferation is inhibited. This threshold can be evaluated using colony forming units of bacteria per milliliter (CFU/mL) to measure inhibition of cell growth. While both survival analysis and resulting probability curves are used to predict the likelihood of a desired outcome (cell death) in the presence of a certain metric (e.g., temperature), they also allow for the use of censored data, which is applicable to ranges of temperature and cell death rates (Hostetler et al., 2021). Censored data is vital to survival analysis as it allows for the inclusion of incomplete data: data where the event of interest (e.g., cell death levels reaching the inhibition threshold) has not yet occurred (Leung et al., 1997; Hostetler et al., 2021). Outcomes resulting in limited cell proliferation at an inexact time are considered left censored, while cell death levels below that result in right censored data (Hostetler et al., 2021).

The resulting injury/non-injury designations at observed values of an independent variable (temperature, cell death rate) can be fit to a distribution (e.g., Weibull). A parametric approach was used in this study because the response of bacteria can be evaluated by relating an input (e.g., temperature or cell death rate) to an outcome (likelihood of cell proliferation). The objective of this study was to demonstrate that survival analysis can provide a predictive model of the impact of elevated temperatures on biofilm-residing bacteria.

## 2 Materials and methods

### 2.1 Experimental approach

As it is a very common material for medical implants, we chose to test photothermal therapy (PTT) on biofilm-coated silicone (Sherertz et al., 1995). Because silicone is not a good absorber of near infrared (NIR) light and would not generate heat well, a second sample of silicone was infused with poly(3,4-ethylenedioxythiophene) hydrate (PEDOT) nanotubes (NT), which are potent photothermal agents (Levi et al., 2012; Vines et al., 2018). Hospital infections involve both Gram-positive (e.g., *S. aureus*) and Gram-negative (e.g., *E. coli*) bacteria. We chose *E. coli* for our biofilm because PEDOT NTs have previously been used for ablation of Gram-negative bacteria (Levi et al., 2012).

### 2.2 Disk materials

To standardize the silicone (SC) and PEDOT NT-infused silicone (PSC) disks, a SYLGARD™ Silicone Elastomer Kit was used as a base. For the PSC disks, 2.5 mg of PEDOT NTs purchased from Sigma Aldrich were added to 3 g of part A of the elastomer kit and stirred until a homogenous mixture was obtained before subsequently adding 2.85 mL of the silicone curing agent (part B). This mixture was then poured into a 60 mm glass dish and cured at 110°C for 4 h. A 5 mm punch-biopsy was used to obtain the disks from the cured material. SC disks were created in the same manner without the addition of the PEDOT nanotubes. Additional information on synthesis, characterization and thermal evaluation of the SC and PSC disks are provided in Rodriguez-Alvarez et al. (2022).

### 2.3 Laser-induced hyperthermia

#### 2.3.1 Thermal measurements

Temperature measurements on the disks were made prior to biofilm growth. Disks were placed on top of 1 cm long, sterile silicone tubes for support in microcentrifuge tubes (MCT), as shown in the inset in Figure 2. Then 750 µL of phosphate buffered saline (PBS) was added to ensure that all disks were submerged at the same depth. The lids from the MCTs were left open, and a K-Cube® laser (Summus Medical Laser Inc.) with a 1 cm beam diameter, 800 nm continuous wavelength (CW), and 3 W power (for a laser power density of 3.82 W/cm^2^) was used for NIR stimulation. It is well accepted that water has an optical absorption minima within the region from 700–900 nm, hence 800 nm was chosen to minimize thermal heating from water (Weissleder, 2001). The laser was applied for 200 sec and measurements were taken in triplicate at 4, 16, 31, 49, 71, and 81 sec using a new disk sample for every trial. Temperatures were recorded in triplicate, immediately after laser exposure, using a fiberoptic probe (Qualitrol Neoptix® and Nomad thermometer), where the fiber optic measured the temperature of the solution, without touching the dick or vessel walls. The biofilm should have negligible absorption because of its thinness and the laser wavelength used. Therefore, the temperatures achieved during the biofilm laser treatment should be the same as those measured when evaluating the SC and PSC disks alone.

#### 2.3.2 Biofilm development and treatment

A culture of *Escherichia coli* CFT 073 (ATCC® 700928™) in Nutrient Broth No.1 (NB1) broth was developed by shaking at 37°C overnight. The broth was diluted to an optical density (OD) of 0.75–0.85 at 600 nm. Twenty-one SC and PSC disks were added to a 48-well plate and 500 μL of the bacterial suspension was added to each disk. The plate was incubated overnight at 37°C to allow biofilm to grow on the disks. Once the biofilm matured (as determined by visual inspection of the disks, which had an thin, white, glossy coating), the SC and PSC samples were placed in MCTs with 750 µL of sterile water and exposed to the 3 W, CW 800 nm laser for 4, 16, 31, 49, 71, or 81 s to induce elevated temperatures on PSC, while SC samples that do not generate heat, were used as controls. After treatment, biofilms were detached by vortexing for 30 s and sonication (using a Branson water bath sonicator for 2 min). Samples were serially diluted down to 10 ^ ^−8^, plated onto NB1 agar plates, incubated at 37°C for 16 h, and the number of colony forming units per mL was determined.

### 2.4 Arrhenius-Eyring-Polanyi (AEP) equation

The resulting bacteria data were used to construct a classical Arrhenius plot to estimate inactivation energy. This was done by estimating the cell death rate *k* (sec^−1^) using experimental bacteria concentrations with and without treatment using the first order kinetics in Eq. 5, where *N* is the amount of *E. coli* (CFU/mL) at time *t* (sec) and *N*_*0*_ is the amount of bacteria prior to treatment (Fujikawa and Itoh, 1998). Eq. 1 was modified to a linear form in Eq. 6 so that, once cell

$$N/N_{0}=e^{-kt}$$ (5)

$$\ln\left(k\right)=\frac{\Delta E_{a}}{R}*\frac{1}{T_{abs}}+lnA$$ (6)

death rate at time *t* had been determined, plotting ln(*k*) and 1/*T*_*abs*_ using the temperatures measured from each disk at the different laser irradiation times, *t*, were used for calculating *k*. *ΔE*_*a*_ and *A* can be obtained by examining the slope of the regression line and *y*-intercept.

For the AEP equation, the *ΔG’* (2,544.8 J mol^−1^), *α* (19.41), and *A*_*AEP*_ (0.007363) values for *E. coli* were obtained by averaging literature values of *E. coli* in Huang et al. (2011); Eq. 2 was used to calculate the cell death rates using temperatures measured from the laser stimulated PSC and SC disks. The natural log of the cell death rates was then plotted against 1/*T* (K^−1^) to achieve an Arrhenius-style plot to look for thermal breakpoints (where a significant change in slope occurs). The presence of these breakpoints shows a non-linear relationship between temperature and cell death rate, which indicates that further predictions using the model may not be accurate.

### 2.5 *D/z*-concept

Using *z,* log *D*_*ref*_ and *T*_*ref*_ data for *E. coli* collected from literature, we used our *T* values to calculate log *D* and *k*_*m*_ through Eqs 3, 4. The *z*-value from literature was 10.6°C and *T*_*ref*_ was set at 70°C (van Asselt and Zwietering, 2006). Van Asselt and Zwietering (2006) provided a mean log *D*_*ref*_ (−0.67 log min), resulting in a mean *D*_*ref*_ of 0.214 min, or 12.8 sec, at 70°C.

### 2.6 Survival analysis

Laser treatment reduced viable bacteria (CFU/mL) at each change in temperature. A threshold at which the biofilm would be considered sufficiently disrupted was selected as having below 1E+06 CFU/mL. The data were reformatted to note the temperature change and cell death rate, and whether the corresponding CFU/mL was under the threshold (“yes”) or not (“no”). The binary output data were then fit to a Weibull distribution per the methods of Hostetler et al. (2021). The Weibull distribution was selected to fit these observations because it yields a characteristic shape that corresponds to observations of how bacteria respond to elevated temperatures: proliferation continuing at small temperature increases, no proliferation at very elevated temperatures, and a transition between that is not well characterized.

All analysis was conducted in RStudio®. We used a custom developed library package consisting of several open-source RStudio® packages from the CRAN repository. The sole use of temperature change can result in incomplete analysis. Since we lack a full temperature history, this process was repeated using cell death rates, calculated using Eqs 3, 4.

## 3 Results

**FIGURE 1:**
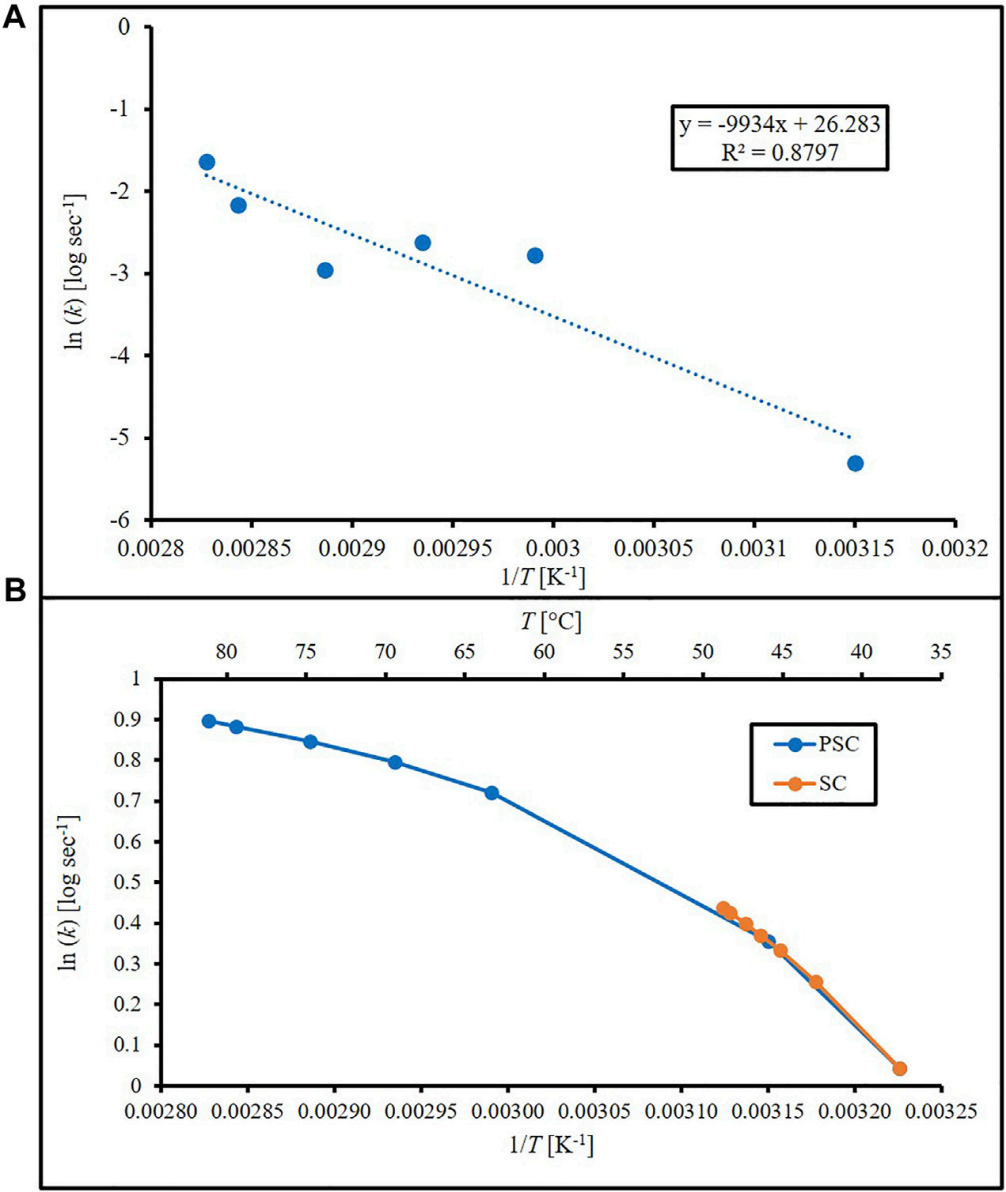
Arrhenius plots (natural log of cell death rate against 1/T) using **(A)** cell death rates calculated from Arrhenius equation using PEDOT-infused silicone (PSC) data with linear regression and **(B)** cell death rates from Arrhenius-Eyring-Polanyi equation and literature constants for silicone (SC) and PEDOT-infused silicone (PSC).

**FIGURE 2:**
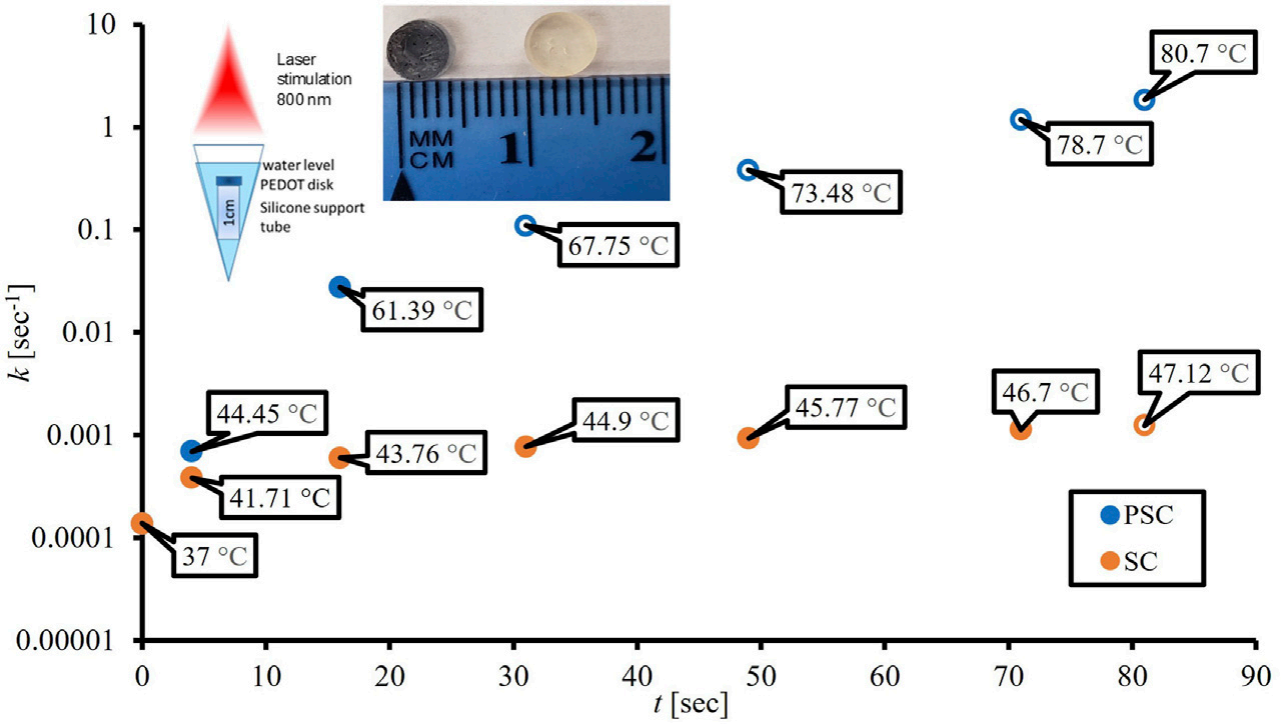
Influence of 3 W laser application times (sec) on cell death rates (sec^−1^) from the *van Asselt and Zwietering* model for silicone (SC) and PEDOT-infused silicone (PSC) with open circles representing points below the 1E+06 CFU/mL survival analysis threshold. From left to right, the first insert image shows the experimental set-up for the disk materials to ensure their orientation, distance from the laser and depth of water covering the disk. The second insert image shows the color differences between silicone (optically transparent) and PEDOT nanoparticle-doped silicone (dark blue).

Table 1 combines the measured temperature changes and CFU/mL values of the different samples and laser stimulation timepoints, as well as standard error of the mean (SEM) values. Temperature *versus* time plots have been published previously, and we direct the reader to see the results in Rodriguez-Alvarez et al. (2022). It also provides the variables required for AEP model calculations, including cell death rate, *k*_*AEP*_ (sec^−1^). Figure 1A is an Arrhenius plot of the PSC data based on Eqs 5, 6. Linear regression resulted in an estimated *ΔE*_*a*_ and A of 82.6 kJ and 2.60E+11 sec^−1^, respectively, but the *R* ^2^ value (0.88) showed little confidence in the linear relationship. Figure 1B is based on the AEP model results and shows two thermal breakpoints for PSC at 44.45°C and 61.39°C. Log *D* (log min), *D* (sec), and *k* (sec^−1^) calculations using our data and the *van Asselt and Zwietering* model are presented in Table 1 alongside the sample’s position above or below the survival analysis 1E+06 CFU/mL threshold. The log *D* (log min) values for some samples were negative because their *D-*values were less than 1 min (60 sec), which follows the rule of logarithms. The PSC data in Table 1 shows that there is a time between 16 sec and 31 sec where the samples crossed the given CFU/mL threshold, while the SC samples do not show this same phenomenon until some point between 71 sec and 81 sec. The SC 81 sec sample was below the threshold despite having a *D*-value of 1.85E+03 s with a *ΔT* of 10.12°C. The PSC 16 sec sample had a shorter *D-*value (83.3 sec), and over double the temperature increase (*ΔT* = 24.39°C), but was still above the threshold. However, the SC sample was irradiated over four times longer. Figure 2 shows the cell death rates (sec^−1^) for biofilms on the PSC and SC disks against laser application time. This plot showcases the difference in cell death rates and temperatures achieved between the PSC and SC samples, as well as the variable heating rate. Cell death rates calculated from the *van Asselt and Zwietering* modified D/z model are sometimes orders of magnitude different from the AEP calculations (Table 1), indicating vast differences between the models for evaluating photothermal inactivation of biofilm-residing bacteria. Even after 81 sec, SC disks could only produce a temperature change of 10.12°C (actual temperature 47.12°C), which resulted in a *D*-value of approximately 31 min (1.85E+03 sec), meaning it would take that long at 47°C to kill 90% of the biofilm-residing *E. coli.* Meanwhile, PSC reached over double that temperature increase (24.39°C) at 16 sec, resulting in a *D*-value of 1.4 min (83 sec). After 31 sec of laser treatment, the PSC reached nearly 70°C, which would need only 21 sec to kill 90% of bacteria. The cell death rate disparities were just as large as the *D*-values. While PSC disks managed an almost 2-log increase in cell death rate at 16 sec (*k* = 1.38E-04 sec^−1^ to 2.77E-02 sec^−1^), and 4-log increase at 81 sec (*k* = 1.19 sec^−1^), SC disks could not manage a 1-log increase in cell death rate until 71 sec of laser application (*k* = 1.38E-04 sec^−1^–1.14E-03 sec^−1^).

**TABLE 1:** Experimental post-treatment measurements alongside AEP model variables, and calculations derived from the *van Asselt and Zweitiering* model and indication of the where the surviving bacteria are above or below the survival threshold of 1E+06 CFU/mL.

(Sample) (Power) (Time)	Mean *ΔT* ± SEM (°C)	Mean CFU/mL ±SEM	*T* (°C)	1/*T*(K^−1^)	*k*_*AEP*_ (sec^−1^)	ln (*k*_*AEP*_)	log(*D*) (log min)	*D* (sec)	*k* (sec^−1^)	Below threshold?
PSC Control	0	7.14E+06 ± 9.82E+01	37	0.00323	1.045	0.0439	2.443	1.66E+04	1.38E-04	No
PSC 3W 4 sec	7.45 ± 0.85	7E+06 ± 2.84E+02	44.45	0.00315	1.428	0.356	1.740	3.30E+03	6.98E-04	No
PSC 3W 16 sec	24.39 ± 1.12	2.63E+06 ± 2.54E+01	61.39	0.00299	2.057	0.721	0.1423	83.3	2.77E-02	No
PSC 3W 31 sec	30.75 ± 2.13	7.53E+05 ± 3.47E+01	67.75	0.00294	2.215	0.795	−0.458	20.9	0.110	Yes
PSC 3W 49 sec	36.48 ± 2.46	5.63E+05 ± 2.40E+01	73.48	0.00289	2.331	0.846	−0.998	6.02	0.382	Yes
PSC 3W 71 sec	41.70 ± 2.89	2.0E+03 ± 1.15E+01	78.70	0.00284	2.421	0.884	−1.491	1.94	1.19	Yes
PSC 3W 81 sec	43.70 ± 3.57	0	80.70	0.00283	2.452	0.897	−1.679	1.26	1.84	Yes
SC 3W 4 sec	4.71 ± 1.02	1.2E+06 ± 2.64E+01	41.71	0.00318	1.293	0.257	1.999	5.98E+03	3.85E-04	No
SC 3W 16 sec	6.76 ± 1.36	2.11E+06 ± 1.13E+02	43.76	0.00316	1.395	0.333	1.806	3.83E+03	6.01E-04	No
SC 3W 31 sec	7.90 ± 1.58	13.2E+06 ± 1.33E+01	44.90	0.00315	1.449	0.371	1.698	2.99E+03	7.70E-04	No
SC 3W 49 sec	8.77 ± 1.66	5.25E+06 ± 5.29E+02	45.77	0.00314	1.490	0.399	1.616	2.48E+03	9.30E-04	No
SC 3W 71 sec	9.70 ± 1.87	4.43E+06 ± 2.12E+02	46.70	0.00313	1.532	0.426	1.528	2.02E+03	1.14E-03	No
SC 3W 81 sec	10.12 ± 2.02	7.3E+05 ± 4.00E+01	47.12	0.00312	1.551	0.439	1.489	1.85E+03	1.25E-03	Yes

The survival analysis data begins in Table 1 where the position of each biofilm response dependent upon the type of disk material is classified as above or below the cutoff threshold of 1E+06 CFU/mL, alongside the cell death rate calculated using the *van Asselt and Zwietering* model. As previously mentioned, Weibull was selected as the distribution function and is shown in Eq. 7 where *λ* and *m* represent the scale (°C) and shape of the curve respectively and *x* represents either temperature change (°C) or cell death rate (sec^−1^). LI is the likelihood of proliferation inhibition and LCP is the likelihood of cell proliferation. Using temperature change and combining Eqs 7, 8 with

$$LI=1-e^{-{\left(\frac{x}{\lambda }\right)}^{m}}$$ (7)

**FIGURE 3:**
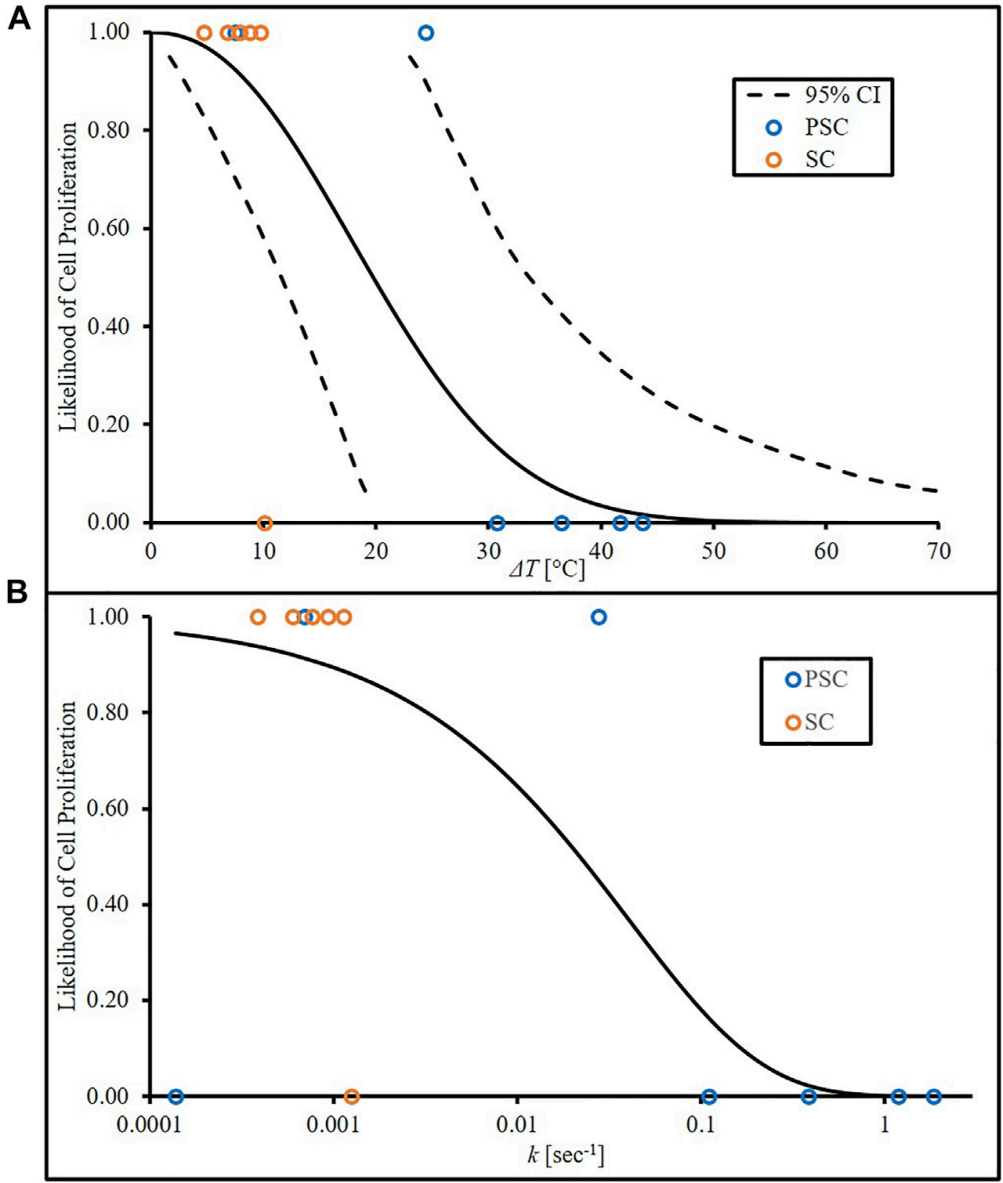
Likelihood of cell proliferation curves from **(A)** temperature increase based on experimental data with lower and upper bounds of a 95% CI and **(B)** cell death rate, *k*, derived from experimental temperature data and *van Asselt and Zwietering* model.

LCP=1−LI (8)

$$LCP_{\Delta T}=e^{-{\left(\frac{x}{23.28}\right)}^{2.236}}$$ (9)

the *λ* and *m* values from the threshold analysis, Eq. 9 becomes the likelihood of cell proliferation dependent upon temperature change (*LCP*_*ΔT*_), with *x* representing temperature change (°C). The parameters of the Weibull model were estimated *via* maximum likelihood estimation model resulting in *λ* and *m* values of 23.28°C and 2.236, respectively (Lee and Wang, 2013). Figure 3A shows this LCP *versus* temperature change, with a 95% confidence interval (CI). This CI represents the range in which the likelihood of cell proliferation can be found at a given level of the independent variable (e.g., temperature). The survival analysis binary data (open circles indicating above or below threshold) are also present to provide a visual representation of how they shape the curves. These were included by applying a value of 1 (above threshold) or 0 (below threshold) to each temperature increase for PSC and SC data. The experimental data showed that, near the limit of mild hyperthermia (45°C; 8°C above body temperature), there was still an 89% likelihood of cell proliferation, and an increase of 38°C would be required to reach 5% likelihood. The cell death rate, *k*, calculations using the new inactivation model were also run through the survival analysis code

$$LCP_{k}=e^{-{\left(\frac{x}{0.04057}\right)}^{0.5945}}$$ (10)

Using cell death rate as *x* in place of temperature increase, resulting in Eq. 10 (*λ* = 0.04057 sec^−1^; *m* = 0.5945), and likelihood of cell proliferation curve dependent on cell death rate (*LCP*_*k*_) was plotted in Figure 3B. Calculated cell death rates were assigned binary values related to the temperature increase used in the calculation. Note that Figure 3A begins at a temperature increase of 0°C and 100% proliferation likelihood. This assumes that there is insignificant cell death at that initial temperature. Values calculated from the *van Asselt and Zwietering* model and input into the survival analysis generate Figure 3B, which accounts for the actual temperature that begins at 37°C. This model assumes that, although minimal, some cell death is expected, therefore likelihood of cell proliferation cannot begin at 100%.

## 4 Discussion

These results align with previous literature that mild hyperthermia (temperatures less than 45°C, but above body temperature of 37°C) is not very effective as a monotherapy for halting proliferation of biofilm-residing bacteria, but ablative temperatures are effective against biofilm. Comparison of the heat generating abilities of SC and PSC with increasing laser stimulation, and thus higher temperatures, provides useful insight for future development of silicone-based photothermal medical materials. Further information of how photothermal nanoparticles for hyperthermia alter biofilm, including altering the structure and biomass, have been provided previously by Yates-Alston, et al. (2021). The PEDOT-imbued silicone (PSC) quickly reached ablative levels that require shorter laser application times to kill the majority of biofilm-residing bacteria, while silicone (SC) required over 2 min of laser application, which the *van Asselt and Zwietering* model predicted would need nearly 20 min of laser application to inactivate 90% of the bacteria. The ability to quickly reach higher temperatures allows for the bacteria to be killed with minimal time for thermal dissipation to surrounding tissue, and hence reduced potential for adverse thermal injury.

The *van Asselt and Zwietering* model reveals a potential flaw in the use of temperature increase as the only variable for bacterial survival analysis. In this case, the initial temperature is not considered and it assumes the likelihood of proliferation is 100% at the initial temperature. The *van Asselt and Zwietering* model avoids this by considering the actual temperature of the sample. With the initial temperature used in this experiment, the use of cell death rate or temperature increase result in similar cell proliferation curves.

Our preliminary data shows survival analysis can be used to determine quantitative models of cell proliferation using temperature change, and related metrics such as cell death rate, as single variables. Since mild hyperthermia may have some effect on disrupting biofilm by reducing viable bacteria at lower temperatures, future studies focused on using survival analysis to model the impact of mild hyperthermia as an adjunctive therapy to antibiotics will be valuable. Survival analysis enables such comparisons and allows for the inclusion of covariates to provide greater specificity predicting injury onset. Future efforts can include antibiotic treatment data as a covariate to evaluate a leftward shift due to the presence of antibiotics (e.g., less likely cell proliferation at lower temperatures).

The presented approach to quantify the likelihood of cell proliferation offers several other benefits from the mathematical modeling standpoint. Firstly, the method is flexible enough to handle data censoring (variability within the experimental data, which often occurs in microbiology). As mentioned previously, a data point may be considered right censored if the CFU/mL count was insufficient to reach a desired threshold. Conversely, the data point can be considered left censored if the CFU/mL observed surpassed the selected threshold and it is unknown if lower temperature increases would be sufficient to achieve such a result. These are typical outcomes from hyperthermia experiments and a technique like quantitative survival analysis that can handle them may be a useful tool. Lastly, the use of the distributions considered above ensures that at zero stimulus there would be 100% of cell proliferation. As seen briefly with cell death rate, this is not always included in other modeling techniques such as logistic regression. This may prove useful when comparing survival curves across varying treatments.

Arrhenius and other models are routinely utilized for predicting tumor response to hyperthermia, with and without adjunctive therapies such as radiation and chemotherapy; however, no model yet exists for predicting the response of pathogenic bacteria to heat in the presence or absence of antibiotics. Given that survival analysis requires only a monotonically increasing independent variable to describe outcomes, the current work offers predictive analyses for new strategies for overcoming biofilms.

## Data Availability

The raw data supporting the conclusion of this article will be made available by the authors, without undue reservation.

## Nomenclature

### Abbreviations

*A*: frequency factor, sec^−1^
***A***_***AEP***_: regression coefficient (AEP model)
AEP: Arrhenius-Eyring-Polanyi (model)
**CFU**/mL: Colony Forming Units per milliliter
CI: Confidence Interval
CW: Continuous Wavelength
*D*: time necessary to achieve 1-log reduction in bacteria, min or sec
***D***_*ref*_: reference D-value at *T*_*ref*_ that is pathogen dependent, min
*k*: cell death rate, sec^−1^
***k***_***m***_: cell death rate, min^−1^
LCP: Likelihood of Cell Proliferation
LI: Likelihood of Inhibition
*m*: shape of Weibull distribution curve
MCT: Microcentrifuge Tube
NIR: near infrared
NT: Nanotubes
OD: Optical Density
PBS: Phosphate Buffered Saline solution
PEDOT: Poly(3,4-ethylenedioxythiophene) hydrate
PSC: PEDOT nanotube-infused Silicone
PTT: Photothermal Therapy
*R*: universal gas constant, 8.314 J mol^−1^
SC: Silicone
SEM: Standard Error of the Mean
*T*: temperature, °C
***T***_*abs*_: absolute temperature, K
***T***_*ref*_: reference temperature in the range of the heating process that is pathogen dependent, °C

### Variables

*z*: temperature increase necessary to reduce D by factor of 10, °C
*α*: exponent coefficient (AEP model)
***ΔE***_***a***_: activation energy, J mol^−1^
*Δ*G’: energy term, J mol-1 (AEP model)
λ: size of Weibull distribution curve, °C or sec^−1^ (dependent on independent variable)

### Subscripts or superscripts

abs: absolute
AEP: Arrhenius-Eyring-Polanyi
m: minute
ref: reference
ΔT: change in temperature
